# The journey of resveratrol from yeast to human

**DOI:** 10.18632/aging.100445

**Published:** 2012-03-12

**Authors:** Silvie Timmers, Johan Auwerx, Patrick Schrauwen

**Affiliations:** ^1^ Top Institute Food and Nutrition (TIFN), 6700 Wageningen, The Netherlands; ^2^ Department of Human Biology, NUTRIM School for Nutrition, Toxicology and Metabolism, Maastricht University Medical Center, 6200 Maastricht, The Netherlands.; ^3^ Laboratory for Integrative and Systems Physiology, Ecole Polytechnique Fédérale de Lausanne (EPFL), 1015 Lausanne, Switzerland

**Keywords:** Resveratrol, SIRT1, sirtuins, calorie restriction, aging, metabolic diseases

## Abstract

The natural polyphenolic compound resveratrol was first discovered in the 1940s. In the recent years, this compound received renewed interest as several findings implicated resveratrol as a potent SIRT1 activator capable of mimicking the effects of calorie restriction, and regulating longevity in lower organisms. Given the worldwide increase in age-related metabolic diseases the beneficial effects of resveratrol on metabolism and healthy aging in humans are currently a topic of intense investigation.

Resveratrol (3,5,4'-trihydroxystilbene) was first isolated from the roots of white hellebore (*Veratum grandiflorum O. Loes*) in 1940 [1], and later, in 1963 from the roots of *Polygonum Cupsidatum*, a plant used in traditional Chinese and Japanese medicine [2]. However, the first real interest in this compound came when in 1992 resveratrol was postulated to explain some of the cardio-protective effects of red wine [3] and was suggested to be an important factor in the French Paradox, a term coined to describe the observation that the French population has a very low incidence of cardiovascular disease, despite a diet high in saturated fat [4]. Five years later, in 1997, Jang and colleagues reported resveratrol to work as a chemo-preventive agent, by the ability to inhibit carcinogenesis at multiple stages [5]. Meanwhile, also anti-inflammatory and anti-oxidant properties were identified for resveratrol (see reference [6, 7] for review). Interest in resveratrol peaked after 2003, when Howitz and colleagues [8] identified resveratrol as a potent SIRT1 activator capable of mimicking the effects of calorie restriction [9, 10] and regulating longevity in lower organisms, by extending lifespan in yeast [8], worms [11], flies (although some controversy remains for this organism [12, 13]) and in short-lived fish [14]. Although there are a considerable amount of data supporting the role for resveratrol in SIRT1-mediated lifespan extension, recent data by Burnett et al. [15], however, suggest that SIRT1 may not increase longevity in *Caenorhabditis elegans* and *Drosophila melanogaster*. Currently the exact role of resveratrol and SIRT1 in longevity is still under debate. What however remains uncontested is that resveratrol does appear to delay or attenuate many age-related chronic diseases in animal models [16]. Given these beneficial effects in animal models, the eventual therapeutic effects of resveratrol merit to be investigated in humans. The first reports of such studies are demonstrating that also in human metabolic diseases, resveratrol may improve overall metabolic health status [17, 18].

## Bio-availability and dosage of resveratrol

Resveratrol is a small polyphenol found in various berries, nuts, and other plant sources [6]. A number of studies have demonstrated that resveratrol and other polyphenols have a very low bioavailability, leading to the concern that many of the beneficial health effects observed in either cells or biochemical assays, may not be achievable in humans due to rapid metabolism [19]. So, one important question - though difficult to answer - is “what dose of resveratrol should be used?”.

The bioavailability and pharmacokinetics of resveratrol has been extensively studied in humans as well as experimental animals. In humans, resveratrol is rapidly taken up after oral consumption of a low dose, with the plasma resveratrol concentration peaking about 30 minutes after consumption [19]. Up to 70% of the ingested resveratrol has been found to be bioavailable in humans based on the levels appearing in the plasma [20], with similar levels being reported for rats (~50%) [21]. Both in rats and humans, resveratrol is suggested to undergo an enterohepatic cycle of metabolism. That is, after being taken up quickly by the enterocytes, resveratrol is metabolized to glucuronide and sulfate conjugates, which are secreted back to the intestine where they may be deconjugated and reabsorbed or excreted in the faeces [20, 21]. The enterohepatic cycle thus reduces the concentration of the free compound reaching the different target tissues in the body. In that respect, the low concentration of resveratrol in the blood is likely explained by the enterohepatic cycle, together with rapid metabolism in the liver. The glucuronide and sulfate conjugates, including disulfates and mixed sulfate-glucuronides, are the major metabolites being formed, apart from dihydroresveratrol [22].

So, it is clear that only a small fraction of the ingested resveratrol reaches the body tissues as resveratrol. Furthermore, the amount of resveratrol ingested from dietary sources, such as red wine and juices (rarely exceeding 5 mg/l), often results in plasma levels that are either not detectable or several orders of magnitude below the micromolar concentrations that are employed *in vitro* (~32 nM – 100 μM) [23]. For example, administration of about 25 mg resveratrol results in plasma concentrations of the free form that range from 1 to 5 ng/ml [24] and administration of higher doses (up to 5 g) increased the plasma resveratrol concentration to about 500 ng/ ml [25].

The low doses of resveratrol observed in the plasma after ingestion are worrisome, as the concentrations used *in vitro* are not reached. However, one must bear in mind that resveratrol is lipophilic, meaning that it mixes very well with lipids including membranes and lipoproteins. Therefore, the tissue resveratrol levels may in fact be higher than what is suggested based on plasma levels. However, some of the biological effects of resveratrol are also observed at a very low concentration [26, 27], bringing forward the idea that resveratrol exerts its major effects on the intestinal tissue, affecting the rest of the body through secondary effects that are indispensible of the plasma levels reached by the compound [6].

In rodent models, the doses employed normally range from as low as 0.1 mg/kg up to 1,000 mg/kg, with even higher or lower doses occasionally being used [6]. Moreover, resveratrol has been show to exert biphasic effects [28]. That is, resveratrol employed at low doses (~ 5 mg/kg/d) has been shown to cause weight gain in mice on a high-fat diet [29], whereas at high doses (~ 400/mg/kg/d), there is marked weight loss [10]. Also, cardioprotective effects of resveratrol that are observed at 2.5 or 5 mg/kg/d are reversed when the dose is increased to 25 or 50 mg/kg/d [30]. These results may suggest that resveratrol acts via multiple target pathways, and as such, calculating the effective *in vivo* concentration of resveratrol or designing new studies based on current literature is challenging. Further experiments are necessary to show whether resveratrol or its metabolites accumulate sufficiently in tissues to account for the widespread acclaimed role of resveratrol in the treatment of various diseases. But even if the optimal dose for rodent experiments is determined, the question still remains how to extrapolate to a human equivalent dose. Direct extrapolation by body weight is often used as a guideline, however, as proposed by others, body surface area might be a better option [31].

## Mechanism of action of resveratrol

The exact mechanisms through which resveratrol exerts a wide range of beneficial effects across species and disease models is currently still unclear [6]. Similar to most other polyphenols, resveratrol is suggested to possess intrinsic anti-oxidant capacity, but it is also implicated to induce the expression of a number of anti-oxidant enzymes, with probably both mechanisms contributing to an overall reduction in oxidative stress [32]. Resveratrol further interacts with a large number of receptors, kinases, and other enzymes that could plausibly make a major contribution to its biological effects.

In 2003 Howitz and colleagues proposed that resveratrol is capable of increasing the deacetylase activity of Sirtuin 1 (SIRT1), a genetic modulator that is part of the health-promoting pathway that is activated by calorie restriction [8]. It has already been known since the 1930s that a severe lowering of calorie intake dramatically slows the rate of ageing in mammals and lowers the onset of numerous age-related diseases, including cancer, cardiovascular disease, diabetes and neurodegeneration [33, 34].

Sirtuins are a conserved family of NAD^+^-dependent deacetylases (class III histone deacetylases) that were named after the founding member, the *Saccharomyces cerevisiae* silent information regulator 2 (Sir 2) protein [35]. A number of subsequent studies showed that resveratrol induced SIRT1 activity in several species (for review see [36]). Furthermore, resveratrol mimics numerous aspects of calorie restriction in all eukaryotes tested to date [8-11, 14, 29, 37] and in most of them, the effect appears dependent on SIRT1 [8, 10, 11]. In line with being an activator of SIRT1, several studies [8, 9, 11, 14], however not all [29], reported that resveratrol increased lifespan. Resveratrol was also shown to increase energy expenditure in mice [9, 10], through increased SIRT1 activation, and during conditions of high fat availability, resveratrol was capable of preventing diet-induced obesity and the onset of obesity-related metabolic diseases, so ultimately protecting mice against the lifespan curbing effects associated with high calorie intake. The molecular mechanism underlying these beneficial effects seem to depend on the resveratrol-induced increase in mitochondrial content, which is explained by increased signalling through the SIRT1/ PGC1α axis [10].

More recently, some studies have questioned the direct activation of SIRT1 by resveratrol [38, 39]. That is, both reports showed that measuring SIRT1 activity by means of the non-physiological fluorescent “Fluor de Lys” substrate assay could lead to artificial results [38, 39]. Furthermore several reports demonstrate that resveratrol can also activate AMPK [9, 40-42], which reconciles with the positive effect on the mitochondrial respiratory chain that has been reported [43]. Hawley et al. [44] reported that resveratrol-induced AMPK activation in isogenic cell lines, stably expressing AMPK complexes containing AMP-insensitive γ2 subunit variants (R531G), derives from an AMP/ATP imbalance as a consequence of interference with mitochondrial respiration. Although the idea was put forward that the resveratrol-induced AMPK activation was dependent on SIRT1 [45], the use of mouse embryonic fibroblast cells from SIRT1 knock-out mice unequivocally demonstrated that SIRT1 is dispensable for resveratrol-induced AMPK activation [40, 46]. On the contrary, resveratrol cannot activate SIRT1 in the absence of functional AMPK [46, 47]. So, the current working mechanism of resveratrol that evolved from all these reports is that SIRT1 functions as the downstream mediator of AMPK, instead of being a direct molecular target of resveratrol. Canto et al. [48] have shown in that respect that the AMPK induced increase in NAD^+^ levels, as a consequence of increased fatty acid oxidation, leads to SIRT1 activation.

Currently, activation of AMPK seems to be a major target of resveratrol's actions, providing a plausible explanation for a large part of the health benefits observed in reports published to date. However, fully defining the targets of resveratrol that are biologically relevant is an enormous task, made more difficult by the question whether effects are either direct or indirect. This issue is discussed further in [6, 7].

## Obesity and diabetes

In rodent models of diet-induced obesity, a high dose of resveratrol (400 mg/kg/d) improves insulin sensitivity and lowers body weight [10], which has increased the interest and the speculation about its potential use as an anti-diabetic agent in humans. However, applying a lower dose of resveratrol (~ 22.5 mg/kg/d) appeared insufficient to produce weight loss, although it still improved glucose tolerance [9]. In fact, low doses of resveratrol are shown to prolong survival in obese mice while simultaneously increasing body weight [29]. One observation that is made, is that animals supplemented with a high dose of resveratrol are capable of increasing their energy expenditure, based on their ability to increase their running distance or tolerate cold longer compared to their untreated controls. However, whether these observations underlie the reduction in body weight is not clear, as voluntary exercise is actually lower in the resveratrol-treated group and body temperature is not detectably changed under basal conditions [10]. Nevertheless, recent work has shown that a one-year intervention with resveratrol at a dose of 200 mg/kg/d seems to cause an increase in basal metabolic rate and total daily energy expenditure in the non-human primate *Microcebus murinus* [49, 50]; indicating that resveratrol might have the potency to enhance energy expenditure thereby promoting weight loss.

In 2007 already, a cross-sectional study found that supplemental resveratrol is taken by 2/3 of people who routinely consume multiple dietary supplements, and this number may be increasing as studies describing resveratrol's health effects have reached the lay public through ample coverage in popular media. Because of the increasing public interest, and the favourable health effects in lower organisms, there is urgent need for studies examining the therapeutic potential of resveratrol in humans, especially since the prevalence of chronic metabolic diseases is reaching epidemic proportions worldwide.

To date, the number of published clinical trials that have examined the effect of resveratrol on insulin sensitivity are still limited and several trials are currently still ongoing (see Table 1 for an overview of all published peer-reviewed clinical trials on resveratrol). However, none of the peer-reviewed human clinical trials have addressed the ability of resveratrol to serve as a weight loss compound. In 2009 a clinical study Elliott et al. [51] reported for the first time the effect of resveratrol on type 2 diabetes patients at doses of 2.5 and 5 g/day for 28 days. The levels of fasting and postprandial glucose and insulin serum levels were statistically significantly decreased at the dose of 5g/day, but few experimental details were provided in that work. In 2011, Brasnyo et al. [52] found that a four-week intervention with resveratrol in type 2 diabetic men significantly improved insulin sensitivity. Thus, supplementing trans-resveratrol twice daily at a dose of 5 mg decreased insulin resistance (computed by the homeostatic model assessment of insulin resistance HOMA-IR), lowered blood glucose levels and delayed the glucose peak following a standardized meal in type 2 diabetic men (n=10) compared with placebo (n=9) [52]. The authors suggested that a decreased oxidative stress might underlie these effects, as significant reductions in 24 h urinary creatinine-normalized ortho-tyrosine concentrations and an increased Akt phosphorylation in blood platelets was observed after the fours weeks of supplementation. Ghamin et al. [53] reported on the other hand that fasting glucose, insulin or HOMA-IR scores remained unchanged following a six-week supplementation of 40 mg resveratrol (in *P. cuspidatum* extract) in healthy volunteers. In that respect it might be noteworthy to mention that also normal healthy mice on a chow-diet fail to improve their insulin sensitivity upon resveratrol supplementation [10], arguing that resveratrol might only be effective under metabolic stress conditions such as obesity or diabetes. Recently, a small pilot study was carried out that showed the potential of resveratrol treatment to improve glucose tolerance, insulin sensitivity and vascular function [17]. For this intervention the authors chose to study the effects of resveratrol in subjects with impaired glucose tolerance that have definite but not yet severe metabolic dysregulation, and therefore may be most amenable to intervention. After four weeks of resveratrol supplementation with a daily dose of 1, 1.5 or 2g, post meal plasma glucose was lowered in IGT subjects at doses between 1 and 2 g/day, whereas the insulin response remained unchanged [17]. Furthermore, a trend towards an improved post meal endothelial function was reported.

**Table 1:** Summary of peer reviewed published clinical trials*

Authors	Participants (n)	Objective	Form and dose of resveratrol	Duration	Outcome
Bioavailability from resveratrol supplement (as capsules or in another matrix)					
**Almeida et al. 2009 [24]**	Healthy men (20) and women (20)	Bioavailability from resveratrol supplement	25, 50, 100, or 150 mg capsules	Multiple, 6x/day at 4h intervals for 13 doses	Peak plasma concentrations of trans-resveratrol were reached at 0.8-1.5 h post dose. Following the 13th dose of trans-resveratrol 25, 50, 100 and 150 mg, mean peak plasma concentration (C(max)) was 3.89, 7.39, 23.1 and 63.8 ng/mL. Interindividual variability was high. Bioavailability was higher after morning administration. Resveratrol was well-tolerated, but with some mild adverse events reported.
**Boocock et al. 2007 [25]**	Healthy men (18) and women (22)	Bioavailability from resveratrol supplement	0.5, 1, 2.5, or 5 g capsules	Single dose	Consumption of resveratrol did not cause serious adverse events. Resveratrol and six metabolites were recovered from plasma and urine. Peak plasma levels of resveratrol at the highest dose were 539 ng/mL, which occurred 1.5 h post-dose. Peak levels of two monoglucuronides and resveratrol-3-sulfate were 3- to 8-fold higher. The AUC values for resveratrol-3-sulfate and resveratrol monoglucuronides were up to 23 times greater than those of resveratrol. Urinary excretion of resveratrol and its metabolites was rapid, with 77% of all urinary agent-derived species excreted within 4 h after the lowest dose.
**Brown et al. 2010 [56]**	Healthy men (22) and women (18)	Bioavailability from resveratrol supplement	0.5, 1, 2.5, or 5 g caplets	Multiple, once daily for 29 days	Plasma C_max_ was 958.6 μg/L following 29 days of 5 g. C_max_ and total AUC for the metabolites dramatically exceeded those for resveratrol. 2.5 g and 5 g caused mild to moderate gastrointestinal symptoms.
**Burkon et al. 2008 [57]**	Healthy males (9)	Bioavailability from resveratrol supplement	85.5 mg of peceid per 70 kg of body weight	Single dose, dissolved in 100 mL of 15% ethanol and made up with a low-fat milk (1.5%) to a total volume of 500 ml	Trans-resveratrol metabolites formed in the plasma and urine were identified and quantified. The metabolites were trans-resveratrol-3-sulfate, trans-resveratrol-3,4-disulfate, trans-resveratrol-3,5-disulfate, trans-resveratrol-3-glucuronide and trans-resveratrol-4-glucuronide. Up to 50% of the plasma trans-resveratrol-3-sulfate, trans-resveratrol-disulfates and trans-resveratrol-glucuronides were bound to proteins.
**La Porte et al. 2010 [58]**	Healthy men (3) and women (5)	Bioavailability of resveratrol supplement	2000 mg capsules; taken with standard breakfast or high-fat breakfast, quercetin (500 mg) or 100 mL 5% alcohol	Multiple, twice daily	Resveratrol in combination with a high-fat breakfast reduced the area under the plasma concentration-time curve and the C_max_ compared to a standard breakfast. Quercetin, or 5% alcohol (100 mL) did not influence trans-resveratrol pharmacokinetics. Resveratrol was well tolerated, although diarrhea was frequently observed.
**Meng et al. 2004 [59]**	Healthy men (3)	Bioavailability of resveratrol supplement	0.03, 0.5, or 1 mg/kg dissolved in 5 mL whisky mixed with 50 mL water	Single	Resveratrol levels were readily detected in the plasma and the urine. The recovery of resveratrol in the plasma suggested a rapid absorption of resveratrol in the gastrointestinal tract.
**Meng et al. 2004 [59]**	Healthy men (3)	Bioavailability of resveratrol supplement	0.32, 0.64, 0.96, or 1.92 mg delivered in grape juice (200, 400, 600, or 1200 mL)	Single	Resveratrol was only detected in the urine at when 600 and 1200 mL of grape juice were given. In grape juice, the level of free resveratrol is rather low. Cis- and trans-Piceid are the major resveratrol derivatives in grape juice.
**Nunes et al. 2009 [60]**	Healthy young men (6) and healthy young women (6) and elderly men (6) and elderly women (6)	Bioavailability of resveratrol supplement	200 mg capsules	Single, followed by multiple doses at 8-hour intervals for 3 days followed by a last single dose at day 4 (total of eight doses of 200 mg)	Pharmacokinetic and metabolite profile. Resveratrol was well tolerated by young and elderly subjects and the kinetic profile was independent of age and gender.
**Patel et al. 2010 [61]**	Colon cancer patients (20)	Bioavailability of resveratrol supplement	0.5, or 1 g/ day	Single dose for 8 days	Trans-resveratrol (674 nmol/g) and resveratrol-3-O-glucuronide (86 nmol/g) were recovered from colonic tissue.
**Ortuno et al. 2010 [62]**	Healthy men (11)	Bioavailability of resveratrol	Randomized, crossover, controlled trial 14 μg/ kg of resveratrol in different matrices: 250 mL red wine, 1 L grape juice, or 10 tablets (red wine extracts enriched with trans-resveratrol)	Single	Plasma trans-resveratrol increased as a response to all grape products and that of cis-resveratrol after wine and grape juice. Despite similar doses of trans-resveratrol being administered, the bioavailability of resveratrol from wine and grape juice is six fold higher than that from tablets.
**Walle et al. 2004 [20]**	Healthy men (3) and healthy women (3)	Bioavailability from 14C-resveratrol supplement	25 mg taken orally and intravenously	Single	Absorption is at least 70% with peak plasma levels of resveratrol and metabolites of around 491 ng/ml and a plasma half-life of 9.2 h. Most of the oral dose was recovered in the urine. Three main metabolic pathways were identified: sulfate and glucuronic acid conjugation of the phenolic goups, and hydrogenation of the aliphatic double bond.
**Goldberg et al. 2003 [19]**	Healthy men (12)	Bioavailability from three different matrices	25 mg/ 70 kg body weight dissolved in 100 mL of white wine (11.5% ethanol), white grape juice, or V8 vegetable juice/ homogenate	Single	Efficient absorption of resveratrol but significant differences in bioavailability pattern between matrices, with plasma resveratrol concentration decreasing most rapidly with V8 and least rapidly using grape juice.
**Gresele et al. 2008 [63]**	Healthy men (9) and women (11)	Bioavailability from moderate wine consumption	300 mL/d intake of red of white wine. Total polyphenolic concentration: Red wine 1.8 g/L; white wine 0.25 g/L	15 days	Plasma resveratrol concentrations increased form 0.72 to 1.33 μmol/L for white wine and from 0.71 to 1.72 μmol/L for red wine.
**Urpi-Sarda et al. 2005 [64]**	Healthy men (11)	Bioavailability from wine consumption	5.38 mg from 250 mL red wine	Single	Resveratrol metabolites were incorporated into low-density lipoproteins after a moderate intake of red wine. The metabolites identified in low-density lipoproteins were trans-resveratrol-3-O-glucuronide, cis-resveratrol-3-O-glucuronide, cis-resveratrol-3-O-glucoside, and free trans-resveratrol.
**Vitaglione et al. 2005 [65]**	Healthy men (14) and women (11)	Bioavailability from wine consumption	3.4, 7.5, or 33 μg/ kg from 300 or 600 mL red wine with three different dietary approaches: fasting, a standard meal, a meal with high and low amounts of lipids	Single	Free trans-resveratrol was found in trace amounts, only in some serum samples collected 30 minutes after red wine ingestion while after longer times resveratrol glucuronides predominated. Trans-resveratrol bioavailability was shown to be independent from the meal or its lipid content. However, Wide the wide variation in subject responses combined with low bioavailability suggests that the combination of polyphenols may account for the French paradox.
**Zamora-Ros et al. 2006 [66]**	Healthy men (10) and healthy women (10)	Bioavailability from wine consumption	0.357, 0.398, or 2.56 mg/day from 300 mL sparkling wine or 200 mL either white wine or red wine	Multiple; once daily, for 28 days	Significant increases in total resveratrol metabolites were observed in the urine after consumption of sparkling, white or red wine. Resveratrol metabolites in urine may be useful biomarkers of wine intake in epidemiological and intervention studies.
**Oxidative stress and inflammation**					
**Ghanim et al. 2010 [53]**	Healthy adults (20)	Oxidative stress and inflammation	Polygonum cupsidatum extract contain 40 mg of resveratrol Randomized, placebo controlled	Daily for 6 weeks	The extract induced a significant reduction in reactive oxygen species generation as shown by a decrease in the expression of P47 (phox), NFκB, JNK-1, PTP-1B, SOCS-3 in mononuclear cells, when compared to placebo and baseline. The extract also suppressed plasma concentrations of TNF-α, IL-6 and CRP.
**Ghanim et al. 2011 [67]**	Healthy men (4) and women (6)	Markers of oxidative stress, inflammation, Nrf-2 binding activity, the concentrations of endotoxin (lipopolysaccharide) and lipoprotein binding protein	Crossover, placebo controlled. - High-fat high-carbohydrate meal with placebo - High-fat high-carbohydrate meal with 100 mg resveratrol and 75 mg grapeskin polyphenols	2 visits, 1 week apart	The supplement containing resveratrol and muscadine polyphenols suppresses the increase in oxidative stress, lipopolysaccharide and lipoprotein binding protein concentrations, and expression of TLR-4, CD14, IL-1β and SOCS-3 in mononuclear cells after a high-fat high-carbohydrate meal. It also stimulates specific Nrf-2 activity and induces the expression of the related antioxidant genes NQO-1 and GST-P1.
**Cardiovascular effects**					
**Kennedy et al. 2010 [68]**	Young healthy men (4) and women (20)	Cognitive performance and localized cerebral blood flow	Double-blind, placebo-controlled, crossover 250 or 500 mg capsules	Single; once daily on 3 separate days	Resveratrol dose-dependently increased cerebral blood flow during task performance, as indicated by total concentrations of haemoglobin. Resveratrol did not enhance cognitive function.
**Wong et al. 2010 [69]**	Overweight/ obese men (14) and post-menopausal women (5) with borderline hypertension	Endothelial function and cardiovascular health	30, 90, or 270 mg in a randomized double-blind crossover design	Each dose for 6 days	Flow-mediated dilation of the brachial artery increased 45 min following 30, 90, and 270 mg doses of resveratrol.
**Cancer**					
**Nguyen et al. 2009 [70]**	Colorectal cancer patients (8)	A phase-I pilot study in which the effects of resveratrol are examined on Wnt signalling in the normal colonic mucosa and colon cancer tissue	4 groups: - N=3: 80 g of grape powder dissolved in water - N=2 120 g of grape powder dissolved in water - N=2 20 mg of resveratrol (capsule containing also quercetin) - N=1 80 mg of resveratrol (capsule containing also quercetin	Daily for two weeks	Grape powder (80 g), which contains low doses of resveratrol in combination with other bioactive components, can inhibit the Wnt pathway in colonic cancer patients but this effect is confined to the normal colonic mucosa.
**Patel et al. 2010 [61]**	Colon cancer patients (20)	Chemo preventive activity	0.5, or 1 g/ day	Single dose for 8 days	Resveratrol reduced tumour cell proliferation by 5%.
**Chow et al. 2010 [71]**	Healthy men (11) and women (31)	Effect on drug- and carcinogen-metabolizing enzymes	1 g caplets	Once daily for 28 days	Resveratrol intervention inhibited the phenotypic indices of CYP3A4, CYP2D6, and CYP2C9 and induced the phenotypic index of 1A2. Overall, GST and UGT1A1 activities were minimally affected by the intervention, although an induction of GST-π level and UGT1A1 activity was observed in individuals with low baseline enzyme level/activity.
**Brown et al. 2010 [56]**	Healthy men (22) and women (18)	Chemo preventive properties	0.5, 1, 2.5, or 5 g caplets	Multiple, once daily for 29 days	Resveratrol decreased circulating IGF-1 and IGFBP-3 in circulating plasma. The decrease was most marked at 2.5 g. The observed decrease might contribute to cancer chemo preventive activity.
**Diabetes, obesity, and metabolism**					
**Elliot et al. 2009 [51]**	Type 2 diabetics	Insulin sensitivity	2.5, or 5 g	Daily for 28 days	Decreased fasting and postprandial glucose and insulin at 5 g.
**Brasnyo et al. 2011 [52]**	Diabetic men (19)	Insulin sensitivity	5 mg capsules	Twice daily for 4 weeks	Resveratrol significantly decreased insulin resistance (as measured by HOMA index), while it increased the pAkt:Akt ratio in platelets. Urinary ortho-tyrosine excretion (a measure of oxidative stress) decreased by resveratrol.
**Timmers et al. 2011 [18]**	Healthy obese men (11)	Metabolic effects	75 mg of resveratrol in a randomized double-blind, placebo-controlled crossover design	Twice daily, for 30 days	Resveratrol improved the metabolic profile: resveratrol reduced sleeping and resting metabolic rate. In muscle, resveratrol activated the AMPK-SIRT1-PGC1α axis. Resveratrol reduced blood glucose and insulin levels, reduced liver fat storage, improved muscle mitochondrial function and reduced inflammation markers in the blood.
**Crandall et al. 2012 [17]**	Older men (3) and women (7) with impaired glucose tolerance	Glucose tolerance and vascular function	1, 1.5, or 2 g	Daily for 4 weeks	Decreased peak glucose and 3-h glucose AUC following a meal at 1.5 and 2 g. Matsuda index for insulin sensitivity improved at 1.5 and 2 g. Trend towards improved hyperemia index.

* This table was partly based on [72, 73].

We also investigated the metabolic effects of resveratrol in obese men [54] and were able to support the notion that resveratrol might have a similar mechanism of action in obese humans as in high-fat fed animals. Supplementation with resveratrol for 30 days induced health effects that were comparable to the effects of calorie restriction. Resveratrol reduced sleeping and resting metabolic rate in the absence of body weight changes. Furthermore, skeletal muscle mitochondrial function and fat oxidative capacity improved and fasting plasma glucose and insulin values were decreased by resveratrol. Gene set enrichment analysis revealed that resveratrol activated similar pathways in humans compared to mice, as mitochondrial pathways related to ATP production and oxidative phosphorylation were upregulated and inflammatory pathways were downregulated. In accordance to the rodent data, we confirmed that resveratrol supplementation induced an increase in skeletal muscle SIRT1 protein levels. These results are especially encouraging since Rutanen et al. showed that low SIRT1 expression could contribute to the disturbance in energy balance, that is already present in offspring of type 2 diabetes, by reducing mitochondrial function [55].

Though limited data is available on resveratrol's efficacy in chronic metabolic diseases in humans, the clinical trials that are available show much promise that resveratrol might be applied to improve general health status and prevent chronic disease in humans. However, further research is warranted to increase our understanding of the physiological responses of resveratrol before widespread use in humans can be promoted. Future research should aim to explore the relationship between dose – bioavailability- and efficacy and further define the pleiotrophic mechanisms of actions in humans. Furthermore, chronic studies are an absolute must, as it is still unclear if resveratrol supplementation on the longer term is beneficial for overall health status.
